# A new feather mite species of the genus *Trouessartia* Canestrini, 1899 (Acarina, Trouessartiidae) – an integrative description (morphology and DNA barcoding data)

**DOI:** 10.3897/zookeys.789.27829

**Published:** 2018-10-10

**Authors:** Ioana Cristina Constantinescu, Oana Paula Popa, Luis Ovidiu Popa, Ioana Cobzaru, Mukhim D. Khlur B., Costică Adam

**Affiliations:** 1 “Grigore Antipa” National Museum of Natural History, Sos. Kiseleff no.1, 011341 Bucharest 1, Romania “Grigore Antipa” National Museum of Natural History Bucharest Romania; 2 Ecology, Taxonomy and Nature Conservation Department, Institute of Biology, Romanian Academy, Splaiul Independenței no. 296, 060031 Bucharest, Romania Institute of Biology, Romanian Academy Bucharest Romania; 3 Department of Zoology, Lady Keane College, 793001 Shillong, Meghalaya, India Department of Zoology, Lady Keane College Shillong India

**Keywords:** Feather mite, new species, taxonomy, *
Trouessartia
*

## Abstract

A new species of the feather mite genus *Trouessartia* (Trouessartiidae) is described from the Large Niltava*Niltavagrandis* (Blyth) (Passeriformes, Muscicapidae) in Northeast India (Meghalaya, Jaintia Hills, Shnongrim village). *Trouessartianiltavae* Constantinescu, **sp. n.** is morphologically closely related (no phylogenetic meaning) to *T.bulligera* Gaud, 1968 from *Clytorhynchushamlini* (Mayr) (Passeriformes: Monarchidae), sharing in males a unique character within the genus, by having setae *e* on legs IV hemispheroid, with spine-shaped apex. Males of the new species have the prodorsal shield without ornamentation, the prohysteronotal shield and lobar shield connected, and the terminal cleft parallel sided. Females have the posterior half of the hysteronotal shield ornamented with large ovate lacunae in central area and small elliptical lacunae marginally. To the morphological description of this new feather mite species we added sequence data on the mitochondrial cytochrome c oxidase subunit I gene fragment (COI). The phylogenetic relationships between *Trouessartia* species are briefly discussed.

## Introduction

The feather mite genus *Trouessartia* Canestrini, 1899 comprises about 120 described species associated predominantly with birds of the order Passeriformes. A world revision of this genus, including 71 species was performed by Santana (1976); other presently known species were described in the subsequent 40 years by various authors (Mauri and de Alzuet 1968, Černý and Lukoschus 1975, Gaud 1977, Černý 1979, Mironov 1983, Gaud and Atyeo 1986, 1987, Mironov and Kopij 1996, 2000, Mironov and Galloway 2002, O'Connor et al. 2005, Carleton and Proctor 2010, Constantinescu et al. 2013, 2016a, 2016b, 2017, 2018, Mironov and González-Acuña 2013, Hernandes 2014, 2017, Hernandes and Valim 2015, Mironov and Overstreet 2016, Mironov and Palma 2016, Mironov and Bermúdez 2017, Hernandes and OConnor 2017). A number of undetermined *Trouessartia* species were reported from the following areas of the world: 22 species from Colombia (Barreto et al. 2012), 15 species from Brazil (Silva et al. 2015) and 162 species from Southeast Asia (McClure and Ratanaworabhan 1973).

The bird genus *Niltava* Hodgson belongs to the family of Old World Flycatchers (Passeriformes: Muscicapidae) and currently includes six valid species distributed in the Indo-Malayan biogeographic region (Clements et al. 2016). Feather mites were previously recorded only on two of these species: *Analges* sp., *Anisodiscus* sp., *Mesalgoides* sp., *Proctophyllodescotyledon* Trouessart, 1899, *Bicentralgesdistinctus* Orwig, 1968, *Proterothrixchachulae* Constantinescu, 2017 and *Trouessartia* sp. from *Niltavagrandis* (Blyth); *Analges* sp., *Proctophyllodeselegans* Atyeo and Braasch, 1966, *Proterothrix* sp., *Therisalges* sp., *Trouessartia* sp., and *Xolalges* sp. from *Niltavasundara* Hodgson (Atyeo 1973, Atyeo and Braasch 1966, Constantinescu et al. 2017, Orwig 1968).

The main goals of this paper are to realise the description of a new species of *Trouessartia* and to analyse its relationships within the genus based on molecular data. This is the first species of *Trouessartia* described from a host of the genus *Niltava*, although, as mentioned above, two presumably new species of *Trouessartia* have been reported by Atyeo (McClure and Ratanaworabhan 1973) from *N.grandis* and *N.sundara*, but they have never been described.

The new species of *Trouessartia* described herein cannot be referred to any of the seven species groups previously established in the genus (Santana 1976, Mironov and Kopij 1996, 2000), because of having a specific combination of characters.

## Materials and methods

The material used in the present paper was collected near Shnongrim (Meghalaya, India) in January 2014. The birds were captured using mist nets, identified and visually checked for the presence and collection of mites and released back to the wild. Mite specimens were collected manually with a needle and placed in tubes with 96% ethanol. Later, in laboratory conditions, mite specimens selected for morphological analysis were cleared in 90% lactic acid for 24 hours and mounted on microscope slides in Hoyer’s medium. Some specimens preserved in ethanol were used for genetic analysis. Drawings were made using an Olympus CX21 microscope, with a camera lucida drawing device. The bird specimens were identified according to Rasmussen and Anderton (2012) and Grimmett et al. (2011), and the taxonomy of the birds follows Clements et al. (2016). The body setation of mites follows that of Griffiths et al. (1990) with the modifications by Norton (1998) concerning coxal setae, while the setation of legs follows Gaud and Atyeo (1996). Description of the new species of *Trouessartia* is given according to the standards proposed for mites of this genus and related genera (Orwig 1968, Santana 1976), and the measuring techniques of particular structures follow Mironov and González-Acuña (2013). We give the full set of measurements for a holotype (male) and a range of measurements for corresponding paratypes. All measurements are in micrometers (μm). The holotype and paratypes of the new species are deposited in the Acarological Collection of the “Grigore Antipa” National Museum of Natural History, Bucharest, Romania (MGAB). The inventory numbers are given in parentheses for all type specimens.

Three paratype specimens of *Trouessartianiltavae* sp. n. (one male ANA838 and two females ANA839, ANA840) were used to isolate DNA using DNAeasy Tissue Kit (Qiagen). All four specimens used for molecular analyses were mounted and kept as reference vouchers for morphological examination. The specimens preserved in ethanol 96% were transferred in 180 μl ATL Buffer with 20 μl of Proteinase K and incubated overnight at 56 °C on a shaking thermoblock. After 24 h, 5μl of Proteinase K were added and incubation was continued until 72 h. For the rest of the protocol we followed the manufacturer specifications and the modification suggested by Dabert et al. (2008).

As DNA barcode we used a region near the 5` terminus of the COI gene, amplified by PCR with the degenerate primers bcdF05 (5`- TTTTCTACHAAYCATAAAGATATTGC-3`) and bcdR04 (5`- TATAAACYTCDGGATGNCCAAAAAA-3`), according to Dabert et al. (2008). The PCR genotyping reaction was performed in a 50 μL total volume containing DNA template, 1X Green GoTaq Flexi Buffer, 2.5 mM MgCl2, each dNTP at 0.1 mM, each primer at 0.5 μM (the primers were M13 tailed) and 1.5 units of GoTaq DNA polymerase (5U/μl) (Promega, Madison, USA). The PCR products were isolated from samples containing visible bands and sent for sequencing to Macrogen (Seoul, South Korea).

Sequence chromatograms were edited and assembled with CodonCode Aligner version 3.7.1. For the phylogenetic analysis we used a dataset comprising three sequences obtained from the new species *Trouessartianiltavae* and 74 sequences belonging to 17 species of *Trouessartia* genus available in GenBank and BOLD data system and four sequences belonging to genus *Calcealges* (see Tabel 1), to be used as outgroup.

MEGA version 7 software (Kumar et al. 2016) was used to identify the most appropriate substitution model, which was subsequently used to generate phylogenetic trees using the Maximum Likelihood (ML) and Neighbor-Joining (NJ) methods. The percentage of replicate trees in which the associated taxa clustered together in the bootstrap test (10000 replicates) was also computed. The same software was used to compute intra-specific pairwise distances between sequences using K2P distance model (Kimura 1980).

## Results

### Family Trouessartiidae Gaud, 1957

#### Genus *Trouessartia* Canestrini, 1899

##### Trouessartia niltavae

Taxon classificationAnimaliaSarcoptiformesTrouessartiidae

Constantinescu sp. n.

http://zoobank.org/4FB1A0C6-E767-4D46-AF88-B3BAB0E4ACD1

**Figure 1.**
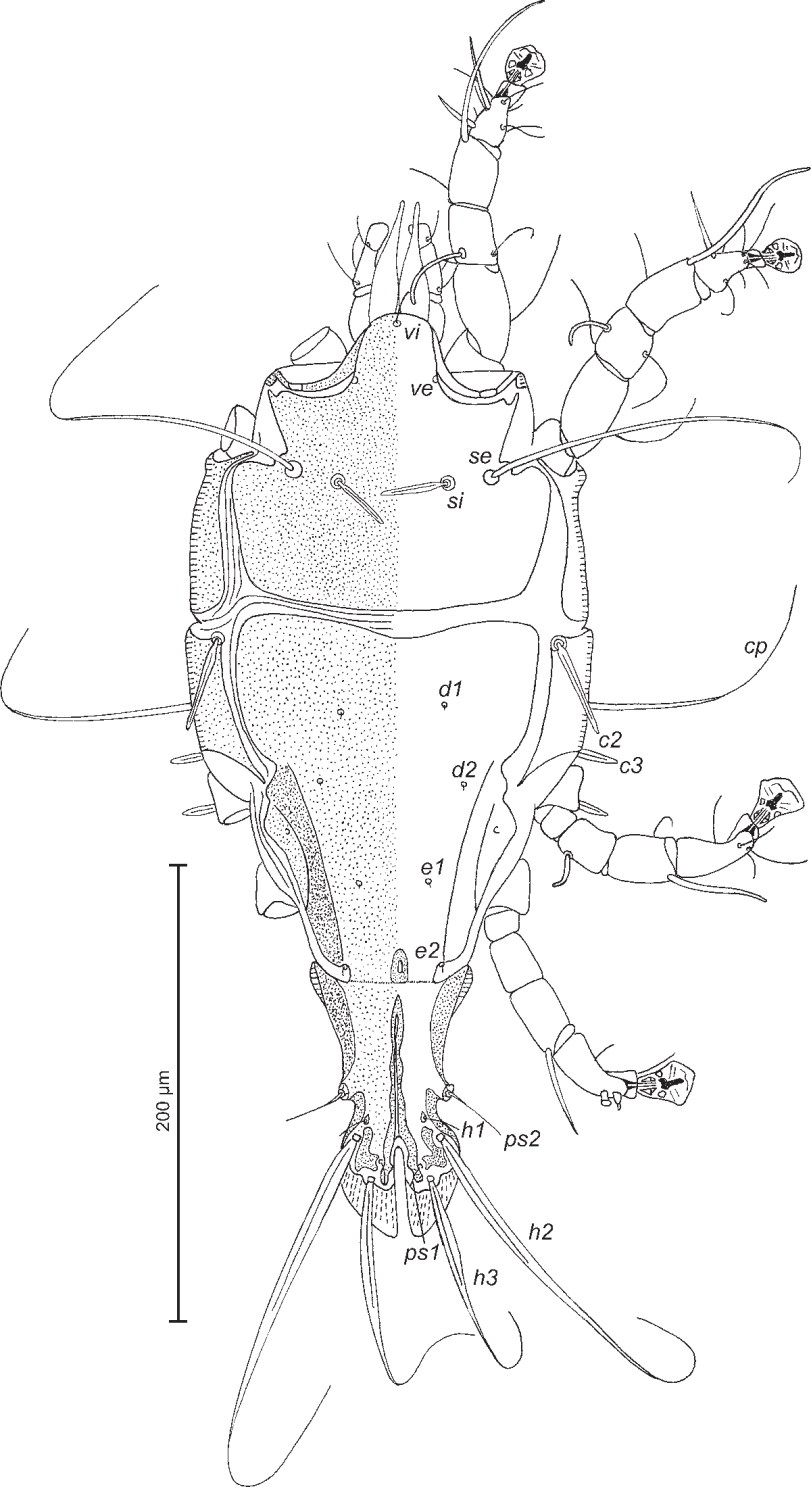
*Trouessartianiltavae* Constantinescu, sp. n., male holotype: dorsal view of idiosoma.

**Figure 2.**
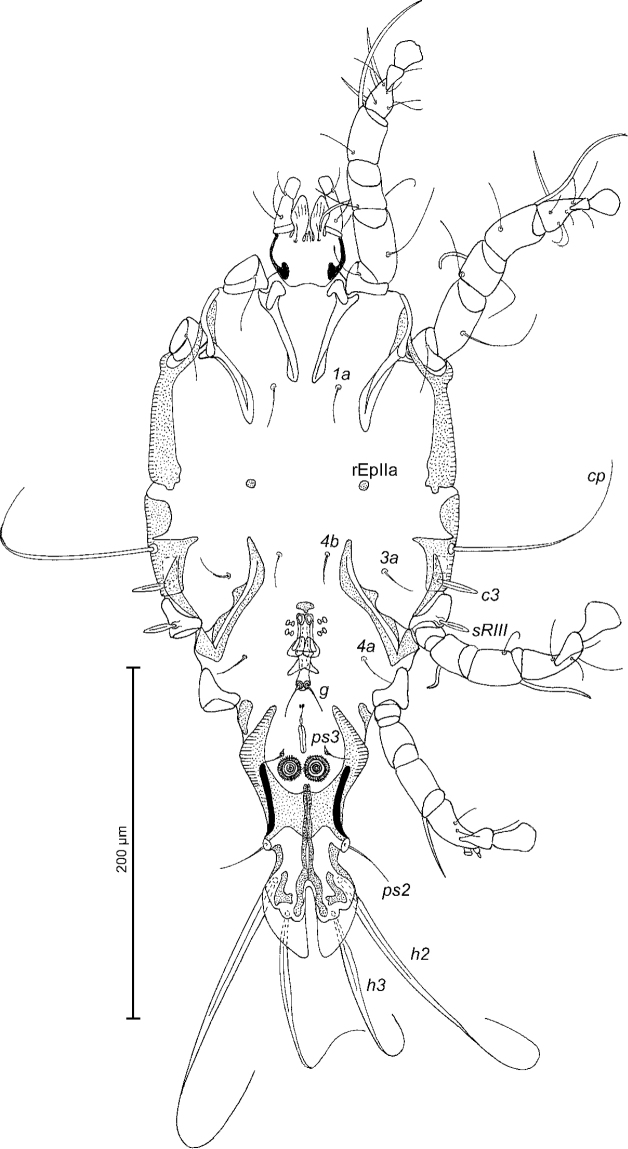
*Trouessartianiltavae* Constantinescu, sp. n., male holotype: ventral view of idiosoma.

**Figure 3.**
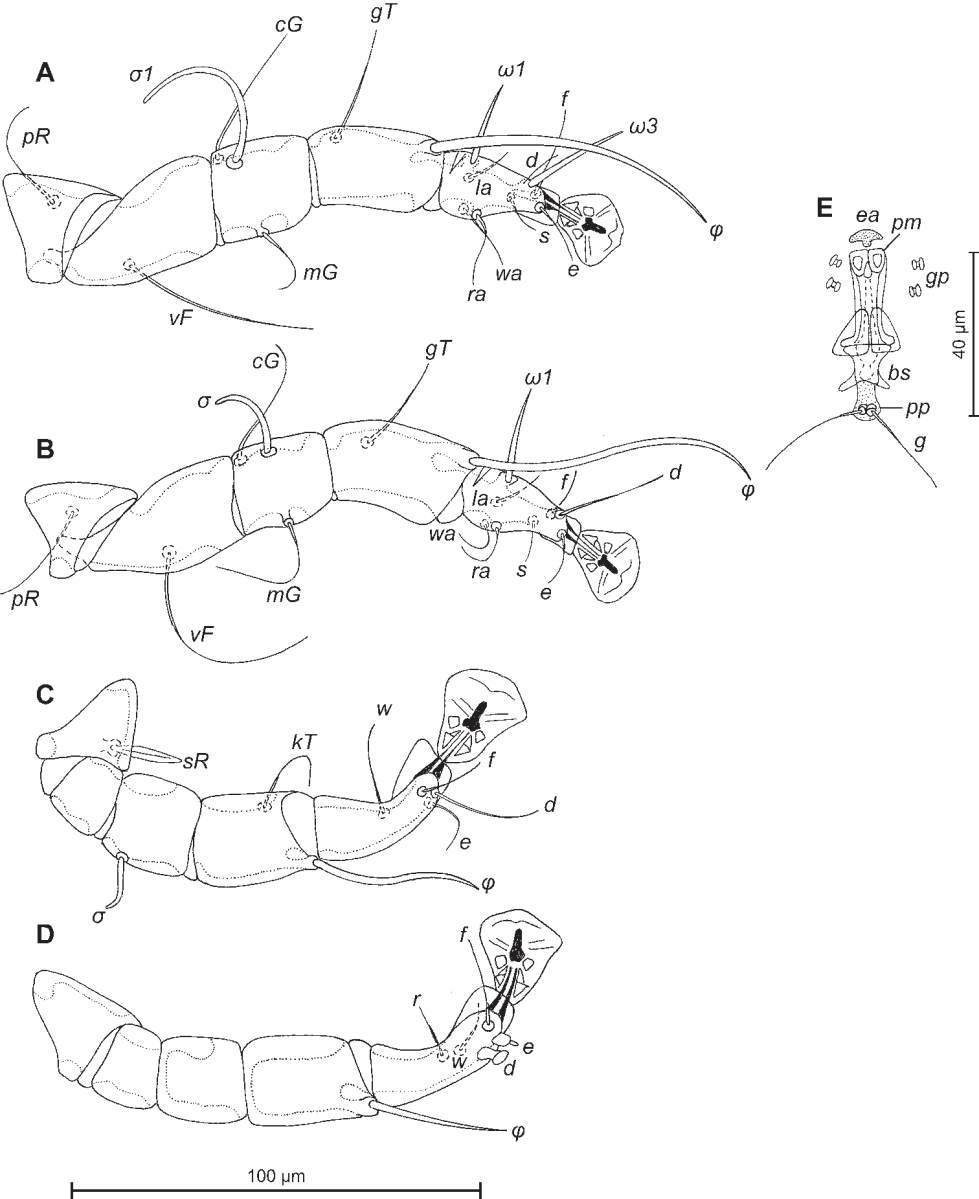
*Trouessartianiltavae* Constantinescu, sp. n., A–D details of male legs, dorsal view: A–leg I, B–leg II, C–leg III, D–leg IV; E–opisthosoma of male, ventral view. Abbreviations: bs – basal sclerite, ea – epiandrum, gp – genital papillae, pm – parameres, pp – postgenital plaque.

**Figure 4.**
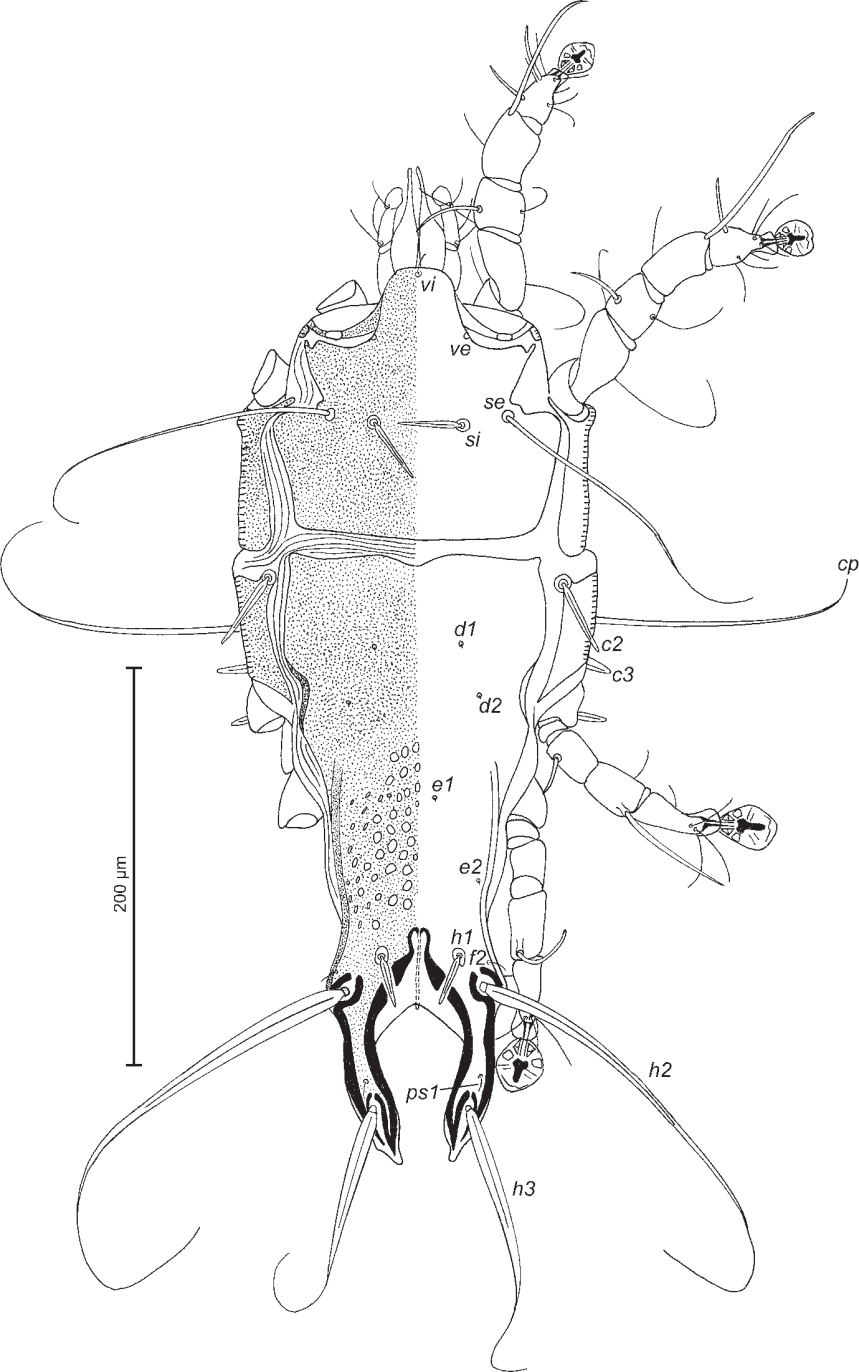
*Trouessartianiltavae* Constantinescu, sp. n., female paratype: dorsal view of idiosoma.

**Figure 5.**
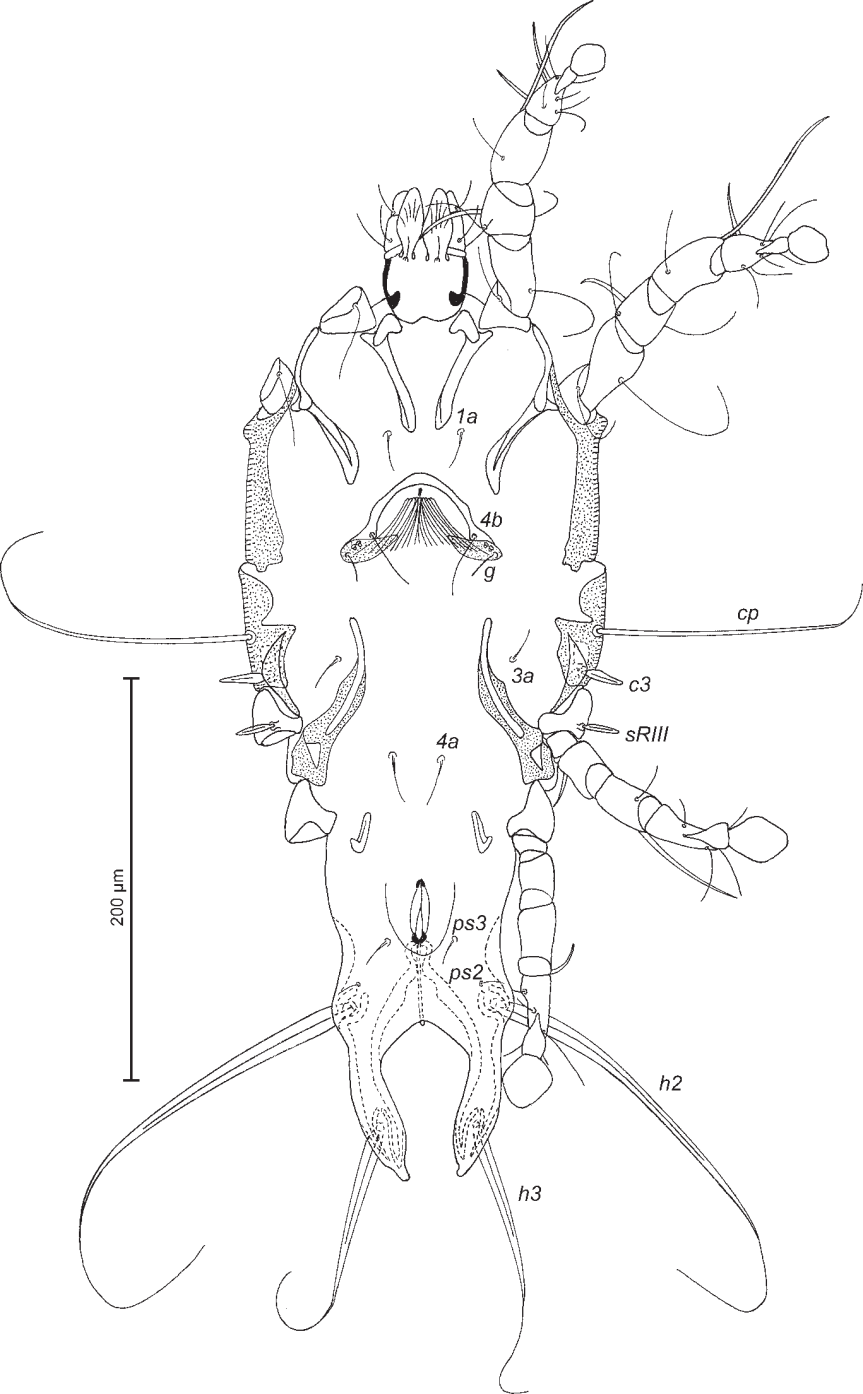
*Trouessartianiltavae* Constantinescu, sp. n., female paratype: ventral view of idiosoma.

**Figure 6.**
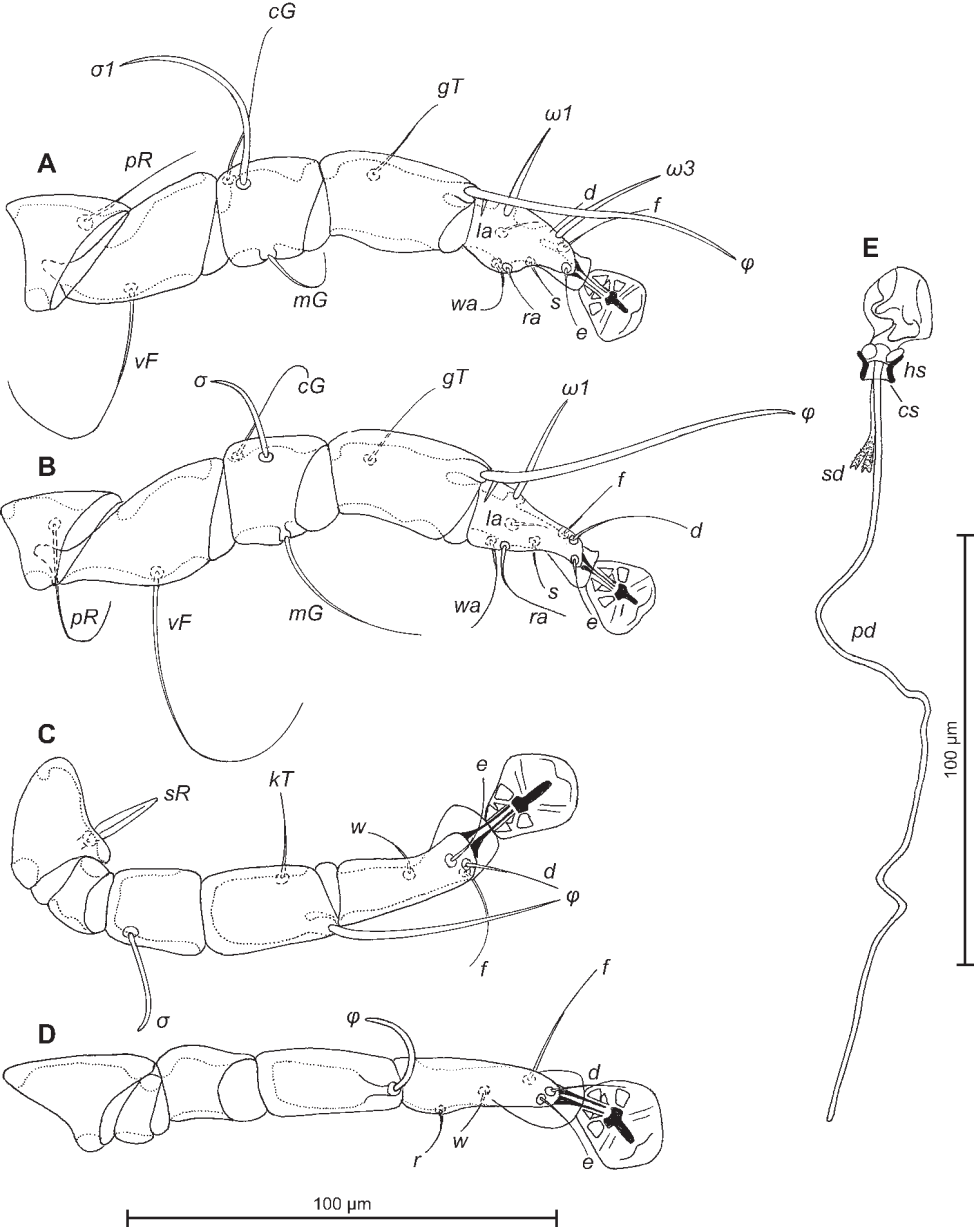
*Trouessartianiltavae* Constantinescu, sp. n., A–D details of female legs, dorsal view: A–leg I, B–leg II, C–leg III, D–leg IV; E–spermatheca and spermaducts of female; Abbreviations: hs – head of spermatheca; pd – primary spermaduct; sd – secondary spermaduct.

Figs 1 , 2 , 3 , 4 , 5 , 6

###### Type material.

Male holotype (ANA663), 5 male (ANA661, ANA662, ANA664, ANA665, ANA838) and 8 female (ANA666, ANA667, ANA668, ANA669, ANA670, ANA839, ANA840) paratypes, 27.01.2014, from the Large Niltava*Niltavagrandisgrandis* (Blyth) (Passeriformes, Muscicapidae); **INDIA**: Meghalaya, Jaintia Hills, Shnongrim village, (25°21’12.36”N, 92°31’3.06”E); 1151 m; subtropical forest; collector D. K. B. Mukhim.

###### Description.

Male (Figs 1; 2; 3A–E; holotype, range for four paratypes in parentheses): length of idiosoma from anterior end to bases of setae *h3* 384 (404–424), greatest width at level of humeral shields 182 (194–202). Length of hysterosoma from sejugal furrow to bases of setae *h3* 250 (260–272). Prodorsal shield: length along midline 130 (130–142), greatest width in posterior part 144 (142–148), lateral margins not fused with scapular shields, antero-lateral extensions almost extending to bases of epimerites Ia between legs I and II, surface without ornamentation (Fig. 1). Internal scapular setae *si* spiculiform, 32 (30–32) long, separated by 48 (42–46); external scapular setae *se* situated on prodorsal shield, separated by 80 (88–93). Vertical setae *ve* represented by alveoli. Humeral shield with setae *c2* spiculiform, 44 (42–46) long. Setae *c3* narrowly lanceolate with acute apex, 23 (20–25) long. Dorsal hysterosoma with prohysteronotal shield and lobar shield connected, delimited from each other by lateral incisions immediately posterior to setae *e2.* Prohysteronotal shield: length 162 (156–162), width at anterior margin 133 (126–132), lateral margins, each with two shallow incisions at level of trochanters III, dorsal hysterosomal apertures (DHA) absent. Dorsal setae *d1*, *d2* present, minute. Length of lobar shield excluding lamellae 90 (90–102). Opisthosomal lobes approximate, separated by narrow parallel-sided terminal cleft; length of this cleft from anterior end to apices of lamellae 42 (44–46), width in anterior part 6 (6–8). Lamellae smooth, slightly attenuate apically, length from bases of setae *h3* to lamellar apices 28 (30–32). Setae *h1* anterior to setae *h2*. Distance between dorsal setae: *c2*-*d2* 80 (80–82), *d2*-*e2* 82 (84–87), *e2*-*h2* 70 (70–76), *h2*-*h3* 22 (20–22), *h2*-*h2* 36 (34–38), *h3*-*h3* 29 (26–28), *d1*-*d2* 30 (24–30), *e1*-*e2* 38 (38–44).

Epimerites I free. Rudimentary sclerites rEpIIa present, roughly rounded. Genital apparatus situated between levels of trochanters III and IV, length excluding basal sclerite 38 (37–40), greatest width 14 (10–14) (Fig. 2). Epiandrum present, setae *g* long and filiform, contiguous at bases, postgenital plaque present. Adanal apodemes heavily sclerotised, with narrow lateral membrane and a pair of apophysis. Translobar apodeme present. Adanal shields very small, teardrop-shaped, bearing setae *ps3*. Anal suckers 12 (12–13) in diameter. Anterior ends of epimerites IV extending beyond level of setae *4b*; epimerites IVa short, not extending to level of genital apparatus. Setae *4b* situated anterior to level of setae *3a*, setae *g* situated posterior to level of setae *4a*. Distance between ventral setae: *4b-3a* 31 (30–36), *4b-g* 72 (74–80), *g-ps3* 48 (44–50), *ps3-h3* 86 (86–94). Setae *sR* of trochanters III narrowly lanceolate, with acute apex 20 (19–24) long, setae *cG* and *mG* of genua I, II filiform, not thickened basally. Tarsus IV 36 (34–36) long; seta *d* barrel-shaped, with discoid cap; seta *e* with hemispheroid base and stick-shaped apical part, situated subapically (Fig. 3D). Legs IV with ambulacral disc extending to level of setae *h3*.

Female (Figs 4; 5; 6A–E; range for five paratypes): length of idiosoma from anterior end to apices of lamellar lobar processes 452–468, greatest width 148–200. Length of hysterosoma from sejugal furrow to apices of lamellar lobar processes 308–324. Prodorsal shield shaped as in male, 130–140 in length, 144–152 in width, surface without ornamentation. Setae *si* spiculiform, 29–34 long, separated by 44–48; external scapular setae *se* situated on prodorsal shield, separated by 90–94. Humeral shields with setae *c2* spiculiform, 42–44 long. Setae *c3* narrowly lanceolate, with acute apex, 22–24 in length. Hysteronotal shield: length from anterior margin to bases of setae *h3* 270–300, width at anterior margin 128–132, lateral margins with shallow concavity at level of trochanters III, bottom of these concavities with dark sclerotisation, DHA absent. Posterior half of hysteronotal shield with distinct ornamentation: with large ovate lacunae in median area and few small elliptical lacunae arranged marginally (Fig. 4). Dorsal setae *d1* and *d2* present. Setae *h1* thick, lanceolate, surrounded by small ovoid area of unsclerotised tegument, 24–28 long, situated antero-mesal to bases of setae *h2*, 20–24 from each lateral margin of hysteronotal shield. Setae *ps1* positioned dorsally on opisthosomal lobes, equidistant from outer and inner margins of lobe, close to bases of setae *h3*. Distance from bases of setae *h3* to membranous apices of lobes 29–32. Setae *f2* present. Supranal concavity open posteriorly into terminal cleft. Length of terminal cleft together with supranal concavity 102–120, width of cleft at level of setae *h3* 38–48. Interlobar membrane occupying anterior 1/3 of terminal cleft, distance from free margin of interlobar membrane to membranous lobar apices 78–88. External copulatory tube minute, 1-2 long, situated on free margin of interlobar membrane. Spermatheca with short collar, primary spermaduct without enlargements, secondary spermaducts with small verrucosities in distal half, length 26–30 (Fig. 6E). Distance between dorsal setae: *c2*-*d2* 76–80, *d2*-*e2* 82–92, *e2*-*h2* 50–56, *h2*-*h3* 58–64, *h2*-*h2* 68–80, *h3*-*h3* 50–60, *d1*-*d2* 29–34, *e1*-*e2* 45–52, *h1*-*h2* 18–21, *h1*-*h1* 38–46, *ps1*-*h3* 9–10.

Epimerites I free. Epigynum 36–38 in length, 76–80 in width (Fig. 5). Epimerites IVa present, short. Setae *sR* of trochanters III narrowly lanceolate, with acute apex, 18–21 long, setae *cG* and *mG* of genua I, II filiform, not thickened basally. Legs IV with ambulacral disc extending to midlevel between setae *h2* and *h3*.

###### Etymology.

The specific name *niltavae* is derived from the generic name of the type host and is a noun in the genitive case.

###### Remarks.

*Trouessartianiltavae* sp. n. is morphologically close to *T.bulligera* Gaud, 1968 from *Clytorhynchushamlini* (Mayr) (Passeriformes: Monarchidae), sharing in males a unique character within the genus: setae *e* on tarsi IV are hemispheroid with stick-like apex. Additionally, in both sexes of these species, setae *d1* are present, setae *c2* and *sR*III are narrowly lanceolate, with acute apex and the dorsal hysterosomal apertures (DHA) is absent. Both sexes of *T.niltavae* differ from those of *T.bulligera* by the shape of setae *c2*, which are spiculiform in the first species versus needle-like in the second. Males of both species have the prohysteronotal shield without ornamentation, the lamellae of opisthosomal lobes are attenuate apically and with entire margins, the translobar apodeme is present, setae *g* are contiguous at bases and situated on postgenital plaque. In males of *T.niltavae*, the prodorsal shield is without ornamentation, the prohysteronotal shield and lobar shield have wide median connection, the terminal cleft is parallel-sided and 44-46 μm long, and terminal lobes are separated by 6–8 μm. In males of *T.bulligera*, the prodorsal shield has ornamentation with faint, interconnecting network of irregular lines, the prohysteronotal shield is completely separated from the lobar shield, the terminal cleft is divergent in posterior half and 75 μm in length, and terminal lobes are separated by 12 μm. In females of both species, setae *h1* are lanceolate, the external copulatory tube is present, the supranal concavity is open posteriorly into terminal cleft, and the interlobar membrane occupies the anterior 1/3 of terminal cleft. In females of *T.niltavae* setae *f2* are present, the posterior half of the hysteronotal shield is ornamented with large ovate lacunae in the central area and small elliptical lacunae marginally arranged. In females of *T.bulligera* setae *f2* are absent, the posterior half of the hysteronotal shield has ornamentation with small elliptical lacunae in the central area and large ovate lacunae marginally arranged.

*DNA barcode.* Representative DNA sequences: molecular voucher specimens ANA838 male (GenBank accession number MH094247), ANA839 female (GenBank accession number MH094248), ANA840 female (GenBank accession number MH094249).

We sequenced a 586-pb fragment of the mitochondrial cytochrome c oxidase subunit I (COI) gene for two females and one male paratypes. All three sequences belong to a single haplotype. The calculated intra-specific genetic distances (K2P) for other species of *Trouessartia* was as follows: *Trouessartiarosterii* 0,8%, *T.reguli* 1,4%, *T.kratochvili* 1%, *T.rubecula* 0,5%, *T.simillima* 0.4%, *T.ripariae* 0,4%, *T.microcaudata* 0,7%, *T.tenuipillata* 0,6%, *T.jedliczkai* 1,6%, *T.trouessarti* 1.4%, *T.motacillae* 1.1%, *T.bifurcata* 11,5%.

**Figure 7.**
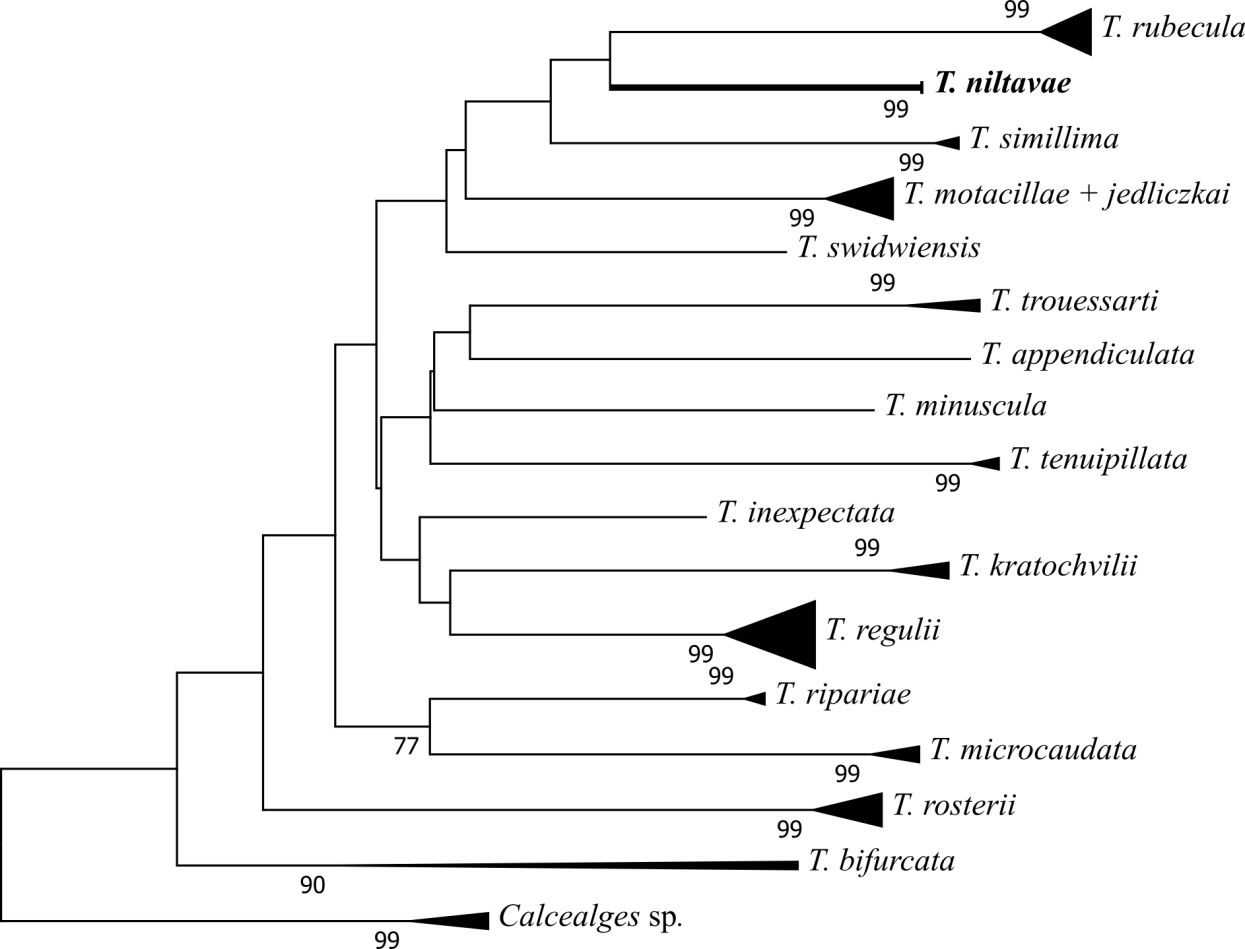
Evolutionary relationship between *Trouessartia* species inferred using the Neighbor-Joining method. The bootstrap test result (10000 replicates) is shown next to each branch.

**Figure 8.**
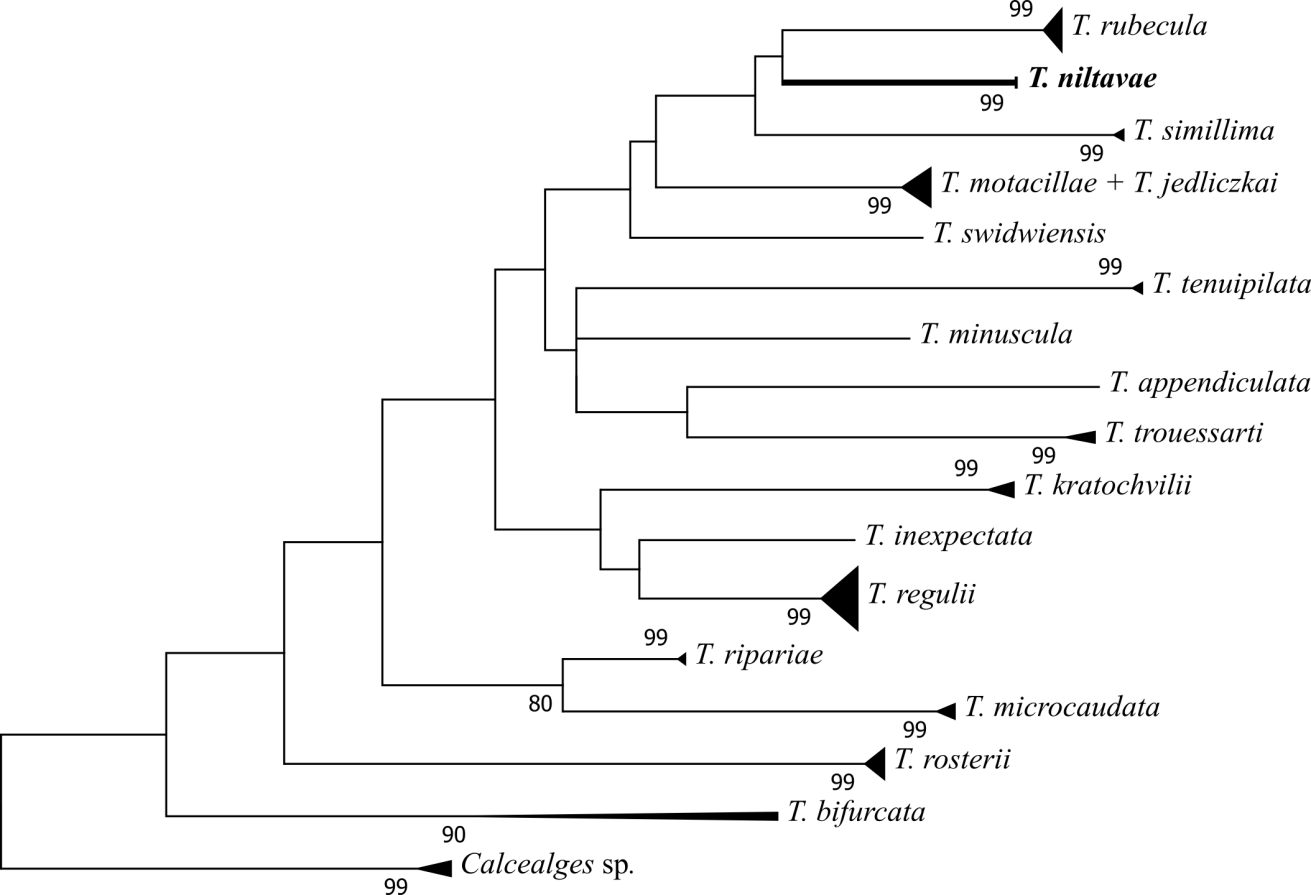
Evolutionary relationship between *Trouessartia* species inferred using the Maximum Likelihood method based on the Tamura-Nei model. The bootstrap test (10000 replicates) shown next to the branches.

The best-fit base substitution model for the analyzed data was determined to be TN93+G+I. The NJ and ML trees exhibited similar topologies and branching structures (see Figures 7 and 8). In both trees our new species was grouped with *Trouessartiarubecula* and *T.simillima*, also described from the Muscicapidae family. Our analysis resolved well the analysed *Trouessartia* species with the exception of *T.motacillae* and *T.jedliczkai*, which were poorly resolved in both analyses. Another noticeable feature of our analysis refers to the species *T.bifurcata*, identified on two avian families: Sylviidae and Acrocephalidae. The two sequences of *T.bifurcata* introduced in our analysis exhibit a high intraspecific diversity which can be a signal of two cryptic species presently identified as *T.bifurcata*.

**Table 1.** Analysed sequences of *Trouessartia* spp. from BOLD and GenBank databases.

**Species**	**GenBank Accession Number**	**Species**	**GenBank Accession Number**	**Species**	**GenBank Accession Number**
* T. appendiculata *	KP193765	* T. rubecula *	KP193799	* T. rosterii *	KP193796
* T. bifurcata *	KP193766	* T. rubecula *	KP193801	* T. rosterii *	KP193797
* T. bifurcata *	KP193767	* T. rubecula *	KP193802	* T. rosterii *	KP193798
* T. inexpectata *	KP193768	* T. rubecula *	KP193803	* T. reguli *	MG411516
* T. jedliczkai *	KP193769	* T. rubecula *	KP193804	* T. reguli *	MG409826
* T. jedliczkai *	KP193770	* T. rubecula *	KP193805	* T. reguli *	MG409631
* T. jedliczkai *	KP193771	* T. rubecula *	KP193806	* T. reguli *	MG414726
* T. jedliczkai *	KP193772	* T. rubecula *	KP193807	* T. reguli *	MG413144
* T. jedliczkai *	KP193773	* T. rubecula *	KP193808	* T. reguli *	MG410216
* T. jedliczkai *	KP193774	* T. rubecula *	KP193809	* T. reguli *	MG412618
* T. kratochvili *	KP193776	* T. rubecula *	KU203092	* T. reguli *	MG411130
* T. kratochvili *	KP193777	* T. simillima *	KP193810	* T. reguli *	MG416767
* T. kratochvili *	KP193778	* T. simillima *	KP193811	* T. reguli *	MG414272
* T. kratochvili *	KU203094	* T. simillima *	KP193812	* T. reguli *	KP193788
* T. microcaudata *	KP193779	* T. swidwiensis *	KP193813	* T. reguli *	KP193789
* T. microcaudata *	KP193780	* T. tenuipilata *	KP193814	* T. reguli *	KP193790
* T. microcaudata *	KP193781	* T. tenuipilata *	KP193815	* T. reguli *	KP193791
* T. microcaudata *	KP193782	* T. tenuipilata *	KP193816	* T. reguli *	KU203095
* T. minuscula *	KP193783	* T. trouessarti *	KP193817	* T. reguli *	KU203096
* T. motacillae *	KP193784	* T. trouessarti *	KP193818	*T.niltavae* sp. n.	MH094247 ^*^
* T. motacillae *	KP193785	* T. trouessarti *	KP193819	*T.niltavae* sp. n.	MH094248 ^*^
* T. motacillae *	KP193786	* T. rosterii *	KT025283	*T.niltavae* sp. n.	MH094249 ^*^
* T. motacillae *	KP193787	* T. rosterii *	KT025284	*Calcealges* sp.	MG410916
* T. ripariae *	KP193792	* T. rosterii *	KT025288	*Calcealges* sp.	MG412689
* T. ripariae *	KP193793	* T. rosterii *	KT025289	*Calcealges* sp.	KU203091
* T. ripariae *	KP193794	* T. rosterii *	KP193795	*Calcealges* sp.	MG409226

* sequences produced in the present study.

## Supplementary Material

XML Treatment for Trouessartia niltavae
